# Comparison Between Oculoplastic Surgeons and Two Large Language Models in the Nonsurgical Management of Thyroid Eye Disease: A Questionnaire‐Based Cross‐Sectional Study

**DOI:** 10.1155/sci5/2900790

**Published:** 2026-10-14

**Authors:** Shiqi Hui, Lihua Luo, Zhijia Hou, Dongmei Li

**Affiliations:** ^1^ Beijing Tongren Eye Center Beijing Tongren Hospital Capital Medical University Beijing China ccmu.edu.cn; ^2^ Department of Ophthalmology Beijing Friendship Hospital Capital Medical University Beijing China ccmu.edu.cn; ^3^ Institute of Digital Ophthalmology and Visual Science Changsha Aier Eye Hospital Changsha Hunan China eye0731.com

**Keywords:** artificial intelligence, clinical decision-making, large language models, nonsurgical management, thyroid eye disease

## Abstract

**Background:**

Thyroid eye disease (TED) presents heterogeneous clinical manifestations requiring nuanced, individualized management. Large language models (LLMs) have shown potential in clinical reasoning, but their consistency with physician decision‐making in nonsurgical TED management remains unclear.

**Methods:**

A structured 19‐item questionnaire covering medical, injection‐based, and radiotherapeutic treatments for TED was distributed to 17 oculoplastic surgeons across the Asia‐Pacific region. Responses were binarized (1 = selected, 0 = not selected) and compared with standardized outputs from GPT and Gemini. Jaccard and cosine similarity indices were calculated to quantify the alignment between physicians and LLMs.

**Results:**

Overall, GPT and Gemini demonstrated comparable alignment with clinicians’ treatment preferences. Both models closely matched physician consensus regarding glucocorticoid use and radiotherapy indications but diverged in immunosuppressant selection and injection‐based strategies. Similarity was generally consistent across physician experience and patient‐volume subgroups.

**Conclusions:**

GPT and Gemini demonstrated moderate agreement with participating oculoplastic surgeons in structured nonsurgical TED management scenarios. These findings support further evaluation of LLM‐assisted applications within controlled clinical validation frameworks, and additional validation using guideline‐based benchmarks and real‐world clinical scenarios is required.

## 1. Introduction

Thyroid eye disease (TED), also known as Graves’ orbitopathy, is a chronic autoimmune inflammatory disorder of the orbit and periorbital tissues associated with thyroid dysfunction. The disease presents with variable activity and severity, ranging from mild eyelid retraction and soft tissue swelling to sight‐threatening dysthyroid optic neuropathy [1, 2]. The management of TED is complex and heterogeneous, often requiring multidisciplinary collaboration between endocrinologists and oculoplastic surgeons.

Nonsurgical management remains the cornerstone of therapy for active or moderate disease and includes glucocorticoid therapy, immunosuppressive agents, biologics, orbital radiotherapy (ORT), and local injection‐based treatments [3, 4]. The optimal timing, dosage, and combination of these interventions, however, remain subjects of clinical debate and are frequently influenced by physician experience, patient preference, and local practice patterns. While international guidelines such as the European Group on Graves’ Orbitopathy (EUGOGO) provide evidence‐based recommendations, substantial variation in real‐world practice persists across regions and clinicians [5].

In recent years, large language models (LLMs) such as OpenAI’s GPT and Google’s Gemini have demonstrated significant progress in clinical reasoning and evidence synthesis [6–8]. Their capacity to generate guideline‐consistent, text‐based therapeutic recommendations has raised growing interest in their potential to support medical decision‐making. Recent studies evaluated multimodal LLMs in thyroid‐associated ophthalmopathy assessment, suggesting AI‐assisted approaches may provide clinically relevant guidance in disease assessment and management for both physicians [9, 10] and patients [11, 12]. Nevertheless, it remains unclear whether LLM‐generated treatment recommendations align with expert clinical judgment in complex, individualized disease contexts such as TED.

To address this question, we designed a structured, questionnaire‐based comparative study to evaluate the degree of concordance between practicing oculoplastic surgeons and two leading LLMs (GPT and Gemini) in the nonsurgical management of TED. We hypothesized that LLMs would demonstrate moderate agreement with practicing oculoplastic surgeons in structured TED treatment scenarios, while differences might emerge in therapeutic domains characterized by greater clinical heterogeneity and individualized decision‐making. By quantifying the similarity between human and AI‐derived therapeutic decisions across multiple treatment domains, this study aims to identify areas of alignment, divergence, and potential clinical integration between artificial intelligence and human expertise in endocrine ophthalmology.

## 2. Methods

### 2.1. Study Design and Participants

This was a cross‐sectional comparative study involving practicing oculoplastic surgeons from Asia‐Pacific medical centers. Between August and October 2024, a structured online questionnaire was distributed to collect data on physicians’ therapeutic decision‐making for TED. Participation was voluntary and anonymous, and all responses were recorded electronically.

### 2.2. Questionnaire Structure

The questionnaire consisted of 31 items (Supporting file 1) designed to capture therapeutic preferences and reasoning across three major domains of nonsurgical TED management: medical therapy (indications, regimens, observed improvements, and adverse events associated with glucocorticoid and immunosuppressive treatments), injection‐based therapy (use of periorbital, intraorbital, or filler injections, including triamcinolone, botulinum toxin, and hyaluronic acid), ORT (indications, timing, observed outcomes, and combination with systemic therapy). This questionnaire was designed based on EUGOGO clinical practice guidelines and commonly encountered real‐world nonsurgical TED management scenarios. Each question permitted multiple selections or self‐statement, which were subsequently standardized into binary form for quantitative analysis. Prior to formal administration, the questionnaire underwent expert review by three senior TED specialists and pilot testing for clarity.

### 2.3. LLM Responses

Two advanced LLMs were included for comparison: GPT (OpenAI, GPT‐5), and Gemini (Google DeepMind, Gemini 2.5 Pro). GPT‐5 (OpenAI) and Gemini 2.5 Pro (Google) were accessed in August 2025 through their publicly available web interfaces. Default model settings were used. Identical prompts were applied across all sessions. Each questionnaire was submitted independently three times. Each LLM was queried using the exact same question wording as in the physician questionnaire. To minimize prompt‐induced bias, all queries were conducted in a consistent format under neutral conditions, without reinforcement or clarification prompts. To evaluate response stability, each question was submitted three times in an independent chat session under identical conditions on the same day. Repeated trials showed consistent outputs, and representative responses were used for downstream analysis. The full LLM responses were independently reviewed by two investigators and transformed into standardized binary variables following the same coding rules used for physician data. Inter‐rater agreement was assessed using Cohen’s kappa coefficient.

### 2.4. Data Encoding and Processing

All categorical variables were encoded as 1 (selected) or 0 (not selected). For each of the treatment‐related items, a binary response vector was generated for each physician and each LLM. Similarity between physicians (*A*) and LLMs (*B*) was evaluated using two complementary indices:

Jaccard similarity coefficient:

(1) JA,B=A∩BA∪B.

Cosine similarity coefficient:

(2) CA,B=A·BAB.

These metrics quantify the degree of overlap and directional agreement between binary vectors, respectively.

To further explore the domain‐level performance of LLMs in TED management, we conducted subgroup analyses within specific therapeutic modules.

### 2.5. Statistical Analysis

Pairwise similarity values were calculated between each physician and each LLM across 31 items. The Wilcoxon signed‐rank test was used to compare mean similarity values between GPT and Gemini. Subgroup analyses were performed by physician experience (< 10 years, 10∼20 years, 20∼30 years, and ≥ 30 years) and total TED case volume (< 100, 100∼500, 500∼1000, and ≥ 1000) using Kruskal–Wallis rank‐sum tests. Descriptive statistics were summarized as mean ± standard deviation. All analyses were conducted using R (version 4.4.2). All statistical tests were two‐sided, and a *p*‐value < 0.05 was considered statistically significant. Violin plot was used to depict the distribution of similarity indices (Jaccard and Cosine) between physicians and LLMs. Heatmap was used to display pairwise similarity matrices (individual physician vs. GPT or Gemini). Bar plot was used to summarize frequency differences across treatment domains (medical, injection, radiotherapy). All plots were generated using R.

## 3. Result

A total of 17 oculoplastic surgeons participated, representing diverse geographic regions and levels of clinical experience. No variation in binary response selections was observed across the three repeated runs performed in this study. The inter‐rater agreement between two investigators in coding LLMs’ answers was high (Cohen’s *κ* = 1.00). The similarity between human clinicians and LLMs was quantified using Jaccard and Cosine indices across all nonsurgical treatment items (Figures 1 and 2 and Supporting Figure 1). The Wilcoxon signed‐rank test indicated no significant difference in the overall concordance between GPT and Gemini when compared with physicians’ responses. Specifically, the median Jaccard similarity between clinicians and GPT did not differ significantly from that of clinicians and Gemini (*V* = 5378.5, *p* = 0.216). Likewise, the Cosine similarity showed no statistically significant difference between the two models (*V* = 5239.5, *p* = 0.432). These findings suggest that GPT and Gemini demonstrated comparable alignment with clinicians’ treatment preferences overall.

**FIGURE 1:**
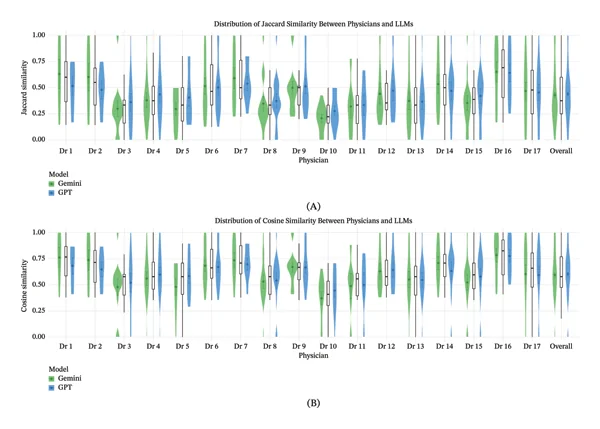
Domain‐specific consistency between ophthalmologists and LLMs across nonsurgical therapeutic modules. Comparison of Jaccard (A) and cosine (B) similarity indices for human–GPT and human–Gemini pairs within each treatment domain. Boxes represent interquartile ranges; dots represent individual respondents. No statistically significant differences were observed between models across domains.

**FIGURE 2:**
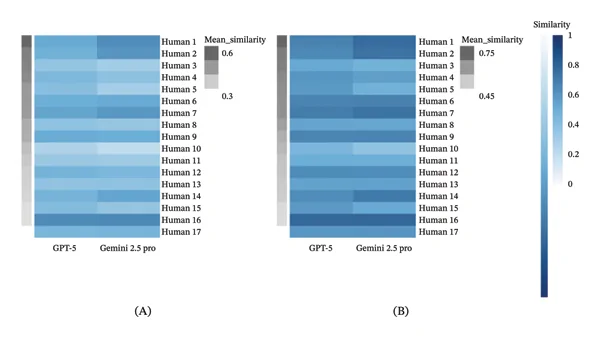
Heatmap of consistency between physicians and LLMs in nonsurgical treatment of TED. Jaccard (A) and cosine (B) similarity coefficients between each physician (rows) and each LLM (columns). Darker colors indicate higher agreement in answer patterns, representing greater consistency between the human and LLM judgments. Physicians are displayed in numeric order to preserve the natural respondent sequence.

### 3.1. Subgroup Analysis by Clinical Experience and Case Volume

To assess whether clinical experience or patient volume influenced concordance with LLMs, physicians were stratified into four subgroups according to their years of clinical practice and the number of TED cases managed in total. Kruskal–Wallis rank sum tests revealed no significant differences in agreement across experience levels or case volume groups for either model (Supporting Table 1).

For GPT, the mean Jaccard similarity did not differ significantly among experience groups (*χ* ^2^ = 2.35, *p* = 0.503) or case‐volume groups (*χ* ^2^ = 4.91, *p* = 0.179). Likewise, Cosine similarity showed no significant difference across experience (*χ* ^2^ = 2.56, *p* = 0.465) or case volume (*χ* ^2^ = 4.66, *p* = 0.199). Similar results were observed for Gemini, with no significant differences by experience (Jaccard: *χ* ^2^ = 1.71, *p* = 0.634; Cosine: *χ* ^2^ = 1.91, *p* = 0.592) or by case volume (Jaccard: *χ* ^2^ = 4.88, *p* = 0.181; Cosine: *χ* ^2^ = 4.44, *p* = 0.218).

These findings suggest that neither seniority nor clinical exposure to TED substantially affected the similarity between clinicians and LLM‐generated treatment recommendations, indicating consistent performance of both models across different professional experience levels.

### 3.2. Subgroup Analysis by Therapy

To further explore the domain‐level performance of LLMs in TED management, we conducted subgroup analyses within specific therapeutic modules.

Within the glucocorticoid therapy domain, no significant difference was observed between Human–GPT and Human–Gemini agreement (Jaccard: *V* = 1203.5, *p* = 0.533; Cosine: *V* = 1170, *p* = 0.849). Similarly, subgroup analyses revealed no significant variation across experience or case volume groups for either GPT (Jaccard: *χ* ^2^ = 3.30, *p* = 0.35; Cosine: *χ* ^2^ = 3.66, *p* = 0.30) or Gemini (Jaccard: *χ* ^2^ = 1.08, *p* = 0.78; Cosine: *χ* ^2^ = 1.04, *p* = 0.79). Notably, the Jaccard similarity between Gemini and physicians showed a marginal difference across case volume categories (*χ* ^2^ = 6.52, *p* = 0.089), suggesting that physicians with higher patient exposure might exhibit slightly more consistent treatment preferences with the model.

Within the immunosuppressive agent’s domain, there was no significant difference in overall agreement between Human–GPT and Human–Gemini responses (Jaccard: *V* = 89, *p* = 0.896; Cosine: *V* = 75, *p* = 0.663). Subgroup analyses also demonstrated no significant differences across experience or case volume groups for GPT (Jaccard: *χ* ^2^ = 4.26, *p* = 0.235; Cosine: *χ* ^2^ = 3.04, *p* = 0.386) or for Gemini (Jaccard: *χ* ^2^ = 7.41, *p* = 0.060; Cosine: *χ* ^2^ = 6.48, *p* = 0.090). Although not statistically significant, the Gemini model exhibited a trend toward higher consistency with more experienced clinicians (*p* ≈ 0.06), suggesting that its treatment recommendations may more closely align with those of experts familiar with immunomodulatory regimens in TED. These findings indicate that both LLMs captured the general therapeutic rationale for immunosuppressive use in TED, but neither model demonstrated domain‐specific superiority.

In the ORT domain, a significant difference was observed between Human–GPT and Human–Gemini agreement (Jaccard: *V* = 181, *p* = 0.004; Cosine: *V* = 180, *p* = 0.005), indicating that GPT’s recommendations were overall more concordant with ophthalmologists than those of Gemini. Subgroup analyses showed no significant variation across experience or case volume strata for either GPT (Experience: *χ* ^2^ = 2.23, *p* = 0.330; Case volume: *χ* ^2^ = 6.76, *p* = 0.080) or Gemini (Experience: *χ* ^2^ = 2.71, *p* = 0.260; Case volume: *χ* ^2^ = 5.00, *p* = 0.172). GPT demonstrated greater concordance within the radiotherapy domain in this dataset.

Orbital injection–based treatment strategies exhibited considerable heterogeneity among respondents and LLMs, reflecting the diversity of clinical experience in periocular (Table 1) and intraorbital (Table 2) corticosteroid administration, as well as Botulinum toxin (Table 3) and hyaluronic acid filler (Table 4) usage. For regimen‐specific questions where nearly every respondent selected unique options, similarity metrics such as Jaccard or cosine were not applicable due to the extremely sparse overlap.

**TABLE 1:** Treatment strategy for periorbital triamcinolone injection of TED.

	Injection site	Therapeutic dose (mg)	Time interval (weeks)	*N* of injections	Side effects during injection
Human 3	Between the superior tarsal border and the superior fornix^∗^^∗^	20	4	2	Eyelid ecchymosis and/or subconjunctival hemorrhage
Human 4	Conjunctiva^∗^	20	4	3	Increase the psychological burden of patients
Human 6	Between the superior tarsal border and the superior fornix^∗^^∗^	20	4	2∼3	/
Human 8	Transcutaneous	40	/	1	/
Human 9	Between the superior tarsal border and the superior fornix^∗^^∗^	40	4	3	Swelling
Human 12	Between the superior tarsal border and the superior fornix^∗^^∗^	40	4	2	/
Human 13	Conjunctiva^∗^	10	/	1	Increased intraocular pressure
Human 15	Conjunctiva^∗^	5	/	1	Increased intraocular pressure
Human 16	Between the superior tarsal border and the superior fornix^∗^^∗^	10	2	3	Eyelid ecchymosis and/or subconjunctival hemorrhage; increased intraocular pressure; Swelling; increase the psychological burden of patients; menstrual changes
Human 17	Conjunctiva^∗^	20	4	3	Increased intraocular pressure
GPT	Between the superior tarsal border and the superior fornix^∗^^∗^	10	2	3	Eyelid ecchymosis and/or subconjunctival hemorrhage; swelling; ptosis; increased intraocular pressure
Gemini	Between the superior tarsal border and the superior fornix^∗^^∗^	20	4	3	Eyelid ecchymosis and/or subconjunctival hemorrhage; increased intraocular pressure; swelling; ptosis; infection

^∗^Conjunctiva just superior to the upper tarsal edge.

^∗∗^The upper eyelid was pulled upward, and the injection was performed between the superior tarsal border and the superior fornix, in the subconjunctival region between the conjunctiva and Müller’s muscle, along the upper border of the tarsus.

**TABLE 2:** Treatment strategy for intraorbital steroid injection for TED.

	Injection site	Therapeutic dose (mg)	Time interval (weeks)	*n* of injections	Side effects during injection
Human 4	Superior temporal quadrant	40	4	3	Increase the psychological burden of patients
Human 9	Superior temporal quadrant	40	2	3	Eyelid ecchymosis and/or subconjunctival hemorrhage, increased intraocular pressure, swelling
GPT	Inferior temporal quadrant	40	2	3	Eyelid ecchymosis and/or subconjunctival hemorrhage, increased intraocular pressure, swelling, ptosis
Gemini	Superior, inferior temporal quadrant, Superior, inferior nasal quadrant	40	4	4	Eyelid ecchymosis and/or subconjunctival hemorrhage, increased intraocular pressure, vision loss, swelling, ptosis, infection

*Note:* GPT: The inferior temporal quadrant is most commonly chosen because it provides a safe access path to the orbital fat, avoids major vessels and nerves, and ensures even drug distribution around extraocular muscles. Gemini: My choice of quadrant depends on the area of maximal muscle inflammation seen on imaging.

**TABLE 3:** Botulinum toxin injection treatment for upper eyelid retraction associated with TED.

	Injection site	Therapeutic dose (UI)	Points per injection	Time interval (months)	Side effects during injection
Human 4	Subconjunctival	5	Two	4	Ptosis
Human 5	Subconjunctival	1.5	Two	6	Diplopia; lagophthalmos and ecchymosis at the injection site; ptosis
Human 9	Subconjunctival	2	Two	3	Ptosis
Human 12	Subconjunctival	5	Three	4	Subconjunctival hemorrhage
Human 15	Subconjunctival	10	Two	3	Ptosis
Human 16	Subconjunctival	5	One	1	Ptosis
Human 17	Subconjunctival	2	Two	3	Ptosis; diplopia
GPT	Transcutaneously	2	Two	3	Diplopia; lagophthalmos and ecchymosis at the injection site; ptosis; subconjunctival hemorrhage; increase the psychological burden of patients
Gemini	Transcutaneously	2∼5	Two	3∼6	Diplopia; lagophthalmos and ecchymosis at the injection site; ptosis; infection

**TABLE 4:** Hyaluronic acid gel filler injection treatment associated with TED.

	Injection site	Therapeutic dose (mL)	Time interval (months)	Side effects during injection
Human 16	Upper eyelid	0.2	3	Erythema, edema, and ecchymosis at the injection site; contour irregularity and lumps, fluid build‐up, and blue or dark color changes; branch retinal artery occlusion
GPT	Upper eyelid; lower eyelid; transcutaneously	0.2	3	Erythema, edema, and ecchymosis at the injection site; contour irregularity and lumps, fluid build‐up, and blue or dark color changes; vison loss; branch retinal artery occlusion; eyelid ecchymosis and/or subconjunctival hemorrhage; increase the psychological burden of patients; infection
Gemini	Upper eyelid; lower eyelid; transcutaneously	0.1∼1.0	6∼12	Erythema, edema, and ecchymosis at the injection site; contour irregularity and lumps, fluid build‐up, and blue or dark color changes; vison loss; branch retinal artery occlusion; eyelid ecchymosis and/or subconjunctival hemorrhage; ptosis; infection

Among respondents, the preferred injection site was between the superior tarsal border and the superior fornix (5/10, 50%), most frequently with 20∼40 mg triamcinolone administered every 4 weeks for 2∼3 sessions. GPT proposed a similar regimen but favored a lower dose (10 mg every 2 weeks), while Gemini recommended 20 mg every 4 weeks, both involving three sessions. Descriptive comparisons suggested broadly similar dose and interval patterns between clinicians and LLMs. Both models correctly recognized eyelid ecchymosis, subconjunctival hemorrhage, and intraocular pressure elevation as common adverse events, consistent with the most common side effects reported by respondents. Gemini was more aligned with typical clinician practices, whereas GPT demonstrated enhanced recognition of clinically plausible side effects.

Respondents consistently selected the superior temporal quadrant (2/2, 100%) as the preferred injection site, using 40 mg triamcinolone at 2 or 4‐week intervals for three sessions. LLMs maintained identical dosing and frequency, but GPT shifted the injection site to the inferior temporal quadrant, whereas Gemini proposed a multidirectional approach across four sessions. Both models identified intraocular pressure elevation, ecchymosis, and swelling as common adverse events; however, Gemini additionally suggested vision loss and infection, reflecting a more comprehensive.

Among respondents, all injections were administered subconjunctivally with therapeutic doses ranging 1.5∼10 UI, most commonly 2∼5 UI applied two points per session. Injection intervals ranged 1∼6 months, with 3∼4 months being most frequent. The most common adverse event was ptosis (6 of 7 respondents), followed by diplopia, lagophthalmos, and subconjunctival hemorrhage. In contrast, both LLMs recommended transcutaneous injection. GPT proposed a uniform dose of 2 UI at two injection points every 3 months, while Gemini suggested 2∼5 UI at two points with intervals of 3∼6 months. LLM‐generated side effect profiles included ptosis, diplopia, lagophthalmos, subconjunctival hemorrhage, and additional events such as infection (Gemini) and increased psychological burden (GPT).

Only one clinician reported performing filler injections for TED‐related eyelid retraction, using 0.2 mL in the upper eyelid every 3 months. Both LLMs extended the indication to upper, lower eyelids, and transcutaneously, with GPT maintaining the same dose and interval, while Gemini proposed a variable 0.1∼1.0 mL dose and 6∼12‐month interval. Descriptive comparison suggested that GPT’s recommendation most closely aligned with actual clinical practice. All sources listed erythema, edema, ecchymosis, and contour irregularity, branch retinal artery occlusion as typical side effects, whereas Gemini included additional rare complications that have been occasionally documented in post‐filler complication reports.

## 4. Discussion

This study evaluated the alignment between oculoplastic surgeons and two LLMs in the diagnosis and management of TED, providing one of the first domain‐specific examinations of LLM performance in a subspecialty clinical context. Therefore, the findings should be interpreted primarily as measures of agreement rather than measures of clinical accuracy. Overall, both GPT and Gemini demonstrated moderate agreement with human clinicians in pattern recognition–based questions, while substantial heterogeneity remained across regimen‐level decisions such as dosing, frequency, and injection‐site preferences. These findings highlight the nuanced interplay between clinician experience, clinical ambiguity, and LLM generalization.

While GPT and Gemini both demonstrated concordance with physicians, statistically differences between the models were observed specifically in ORT. However, given the exploratory nature of this pilot study and its limited sample size, these findings should not be interpreted as evidence of formal equivalence or noninferiority. TED management frequently involves complex and high‐risk decisions that extend beyond binary treatment selection. In real‐world practice, even relatively small discrepancies may carry clinically significant consequences in sensitive scenarios such as disease activity, dysthyroid optic neuropathy recognition, intravenous glucocorticoid dosing, or indications for ORT and biologic therapy. Subtle variations in specific treatment domains may still exist, which warrant domain‐specific analysis rather than global similarity indices alone. The lack of significant subgroup differences implies that the divergence between human and LLM therapeutic reasoning is not primarily driven by clinical seniority or case exposure. This observation aligns with prior studies suggesting that LLMs tend to emulate general patterns of medical decision‐making rather than individual expertise nuances [13–15].

Physicians and LLMs showed high concordance for glucocorticoid therapy in indications, regimen selection, expected improvements, and adverse event recognition. These decisions map closely to guideline‐based algorithms, explaining the strong alignment. Physicians had a wide response heterogeneity among immunosuppressive strategies, likely reflecting variability even among clinicians. Injection‐based treatments showed greater interindividual variability, while both GPT and Gemini captured the general therapeutic rationale, GPT’s recommendations tended to be conservative and closer to the modal clinical patterns, whereas Gemini generated more diverse multisite and multidose regimens. LLMs demonstrated the lowest human–AI similarity in periorbital injection therapies. GPT exhibited more conservative dosing patterns, whereas Gemini offered broader dose ranges and occasionally introduced less commonly emphasized risks such as infection. Human respondents showed substantial variation in therapeutic regimens, particularly in botulinum toxin injection strategies, where choices regarding dose and interval were highly individualized. This inherent heterogeneity may partly explain the limited discriminative value of similarity metrics, particularly for regimen‐specific questions with sparse consensus.

This study offers several strengths. It represents a multinational sample of practicing oculoplastic surgeons, incorporates two advanced LLMs for head‐to‐head comparison, and applies both global and domain‐specific analyses to capture the multidimensional nature of clinical decision‐making. Additionally, the study examined agreement between LLM‐generated responses and real‐world expert practice patterns, providing insight into how closely current LLMs reflect contemporary clinical reasoning in TED management.

However, limitations must be acknowledged. First, this study evaluated concordance rather than clinical accuracy. No external gold‐standard reference, such as formal EUGOGO guideline scoring or expert‐panel adjudication, was used to determine whether physician or LLM responses were objectively correct. Consequently, the findings should be interpreted as measures of alignment with expert‐reported practice patterns rather than evidence of clinical appropriateness or superiority. Second, the sample size was relatively small (*n* = 17) and primarily drawn from the Asia‐Pacific region. In addition, several treatment modalities, including intraorbital steroid injection and hyaluronic acid filler injection, were represented by only a small number of respondents. Consequently, findings related to these domains should be interpreted cautiously and require validation in larger cohorts. Although participants represented multiple countries, TED management practices may differ substantially across healthcare systems because of variations in biologic availability, ORT utilization, reimbursement policies, and local treatment protocols. Therefore, the generalizability of these findings to other geographic regions remains uncertain. Third, the evaluated LLM outputs reflected model versions available at the time of the study. Each questionnaire was independently submitted three times and yielded consistent binary response selections; however, broader assessments of response stability across model updates, prompting strategies, and testing environments could be analyzed in further study. Fourth, the binary encoding strategy enabled standardized quantitative comparison across physicians and LLMs but inevitably reduced the granularity of complex clinical recommendations. Differences in dosage, treatment interval, injection technique, and individualized management considerations may not have been fully captured, potentially leading to overestimation of agreement in certain treatment domains. Fifth, similarity metrics such as Jaccard and cosine similarity may be less suitable for highly individualized treatment questions, limiting interpretability in regimen‐level comparisons. Alternative approaches, including expert‐panel scoring, guideline‐adherence assessment, Delphi consensus methodology, Cohen’s or Fleiss’ agreement statistics, and real‐world case‐based validation, may provide complementary perspectives in future studies. Given the exploratory pilot‐study design and limited sample size, formal estimation of effect sizes and confidence intervals was not prioritized and should be incorporated in future larger‐scale validation studies. Sixth, this study did not evaluate hallucinations, unsupported recommendations, omission of critical management steps, or potential patient safety risks. These aspects represent clinically important dimensions of LLM performance and warrant dedicated investigation before implementation in high‐stakes clinical environments. Finally, scenario‐based responses do not fully capture the contextual richness of real clinical encounters, where patient‐specific factors, comorbidities, disease severity, and clinician judgment collectively influence therapeutic decision‐making.

Despite these limitations, the findings suggest that current LLMs can generate management recommendations that demonstrate moderate concordance with expert‐reported TED practice patterns. LLMs already demonstrate competence in summarization, staging guidance, and outlining standard treatment options. However, further validation against guideline‐based standards, expert consensus frameworks, and real‐world clinical scenarios is required before broader clinical adoption can be considered. With continued refinement, including domain‐specific fine‐tuning, incorporation of real‐world clinical data, and robust safety guardrails, LLMs may evolve into reliable adjuncts for decision support, patient education, and standardization of care.

## 5. Conclusion

GPT and Gemini demonstrated moderate agreement with participating oculoplastic surgeons in structured nonsurgical TED management scenarios. However, such agreement should not be interpreted as evidence of clinical correctness, patient safety, guideline adherence, or readiness for autonomous clinical use. As LLM capabilities continue to evolve, further evaluation using guideline‐based benchmarks, expert consensus frameworks, and real‐world clinical validation studies will be necessary to establish their clinical reliability and safety. These findings support further evaluation of LLM‐assisted applications within controlled clinical validation frameworks and may inform future efforts toward the responsible integration of AI into oculoplastic practice.

## Author Contributions

Shiqi Hui: conceptualization, data curation, formal analysis, investigation, methodology, visualization, and writing–original draft.

Lihua Luo: methodology and writing–review and editing.

Zhijia Hou: methodology, supervision, and writing–review and editing.

Dongmei Li: conceptualization, investigation, methodology, resources, supervision, and writing–review and editing.

## Funding

This research was supported by the National Natural Science Foundation of China (82071005) and Beijing Natural Science Foundation (L2604047).

## Disclosure

All authors had full access to all the data in the study and take responsibility for the integrity of the data and the accuracy of the data analysis.

## Ethics Statement

The Ethics Committee of Capital Medical University Afflicted Beijing Tongren Hospital approved the study (TRECKY2018‐056‐GZ(2022)‐07).

## Conflicts of Interest

The authors declare no conflicts of interest.

## Supporting Information

Additional supporting information can be found online in the Supporting Information section.

## Supporting information


**Supporting Information 1** Supporting Table 1. Characteristics of participating physicians. Supporting Figure 1. Overall consistency between physicians and LLMs.

**Supporting Information 2** Supporting file 1. Original questionnaire used for this multinational survey.

## Data Availability

The data that support the findings of this study are available in the supporting information of this article.
