# Supplementary material for: Comparison Between Oculoplastic Surgeons and Two Large Language Models in the Nonsurgical Management of Thyroid Eye Disease: A Questionnaire‐Based Cross‐Sectional Study
Source: Scientifica (Cairo). 2026 Oct 14;2026:2900790. doi: 10.1155/sci5/2900790 (PMC13669656; doi:10.1155/sci5/2900790)
Supplement: Supplementary file 1 [file SCI5-2026-2900790-s002.pdf]

Preferred Practice Patterns in TAO

Demographics and training background:

Where is the country of your clinical practice?
*

- ◯ Australia & New Zealand
- ◯ Bangladesh
- ◯ China’s mainland
- ◯ Hong Kong
- ◯ India
- ◯ Indonesia
- ◯ Irania
- ◯ Japan
- ◯ Malaysia
- ◯ Mongolia
- ◯ Nepal
- ◯ North Korea
- ◯ Pakistan
- ◯ Philippines
- ◯ Singapore
- ◯ South Korea
- ◯ Taiwan
- ◯ Thailand
- ◯ Others (Please Specify) ________________

Which sector is your clinical practice in (tick as many as applicable)?*

- [ ] Private
- [ ] Public
- [ ] Academic Institution

How many years have you been practicing in your sub-specialized in Oculoplastic surgery for? *

- ◯ <10 years
- ◯ 10~20 years
- ◯ 20~30 years
- ◯ >30 years

Where did you receive your oculoplastic training (tick as many as applicable)?*

- ☐ Australia & New Zealand
- ☐ Bangladesh
- ☐ Canada
- ☐ Cambodia
- ☐ China’s mainland
- ☐ Europe
- ☐ Hong Kong
- ☐ India
- ☐ Japan
- ☐ Malaysia
- ☐ Mongolia
- ☐ Nepal
- ☐ North Korea
- ☐ Pakistan
- ☐ Philippines
- ☐ Singapore
- ☐ South Korea
- ☐ Taiwan
- ☐ Thailand
- ☐ United Kingdom
- ☐ United States
- ☐ Other (Please Specify) ______________________

On average, how many new cases of TED do you manage per year?*

- ○ <5
- ○ 5~10
- ○ 10~50
- ○ 50~100
- ○ >100

How many total cases of TED have you managed in your career thus far? *

- ○ <10
- ○ 10~100
- ○ 100~500
- ○ 500~1000
- ○ >1000

Do you treat TED with medical treatment?*

- ◯ Never
- ◯ Sometimes
- ◯ Always

When do you recommend medical treatment for TED patients (tick as many as applicable)?*

- ☐ Early stages of TED
- ☐ Recurrence of TED
- ☐ Active phase of TED
- ☐ Inactive phase of TED
- ☐ Mild TED
- ☐ Moderate to Severe TED
- ☐ Rapidly progressive, including patients with significant motility deficits
- ☐ DON
- ☐ Corneal involvement
- ☐ Patients with progressive strabismus
- ☐ Congestive symptom
- ☐ Proptosis
- ☐ Eyelid retraction
- ☐ Lower eyelid entropion
- ☐ Diplopia
- ☐ Enlargement of extraocular muscles
- ☐ Cosmesis
- ☐ Other (Please Specify) ________________________

What medical treatment do you use to treat TED? *

- [ ] Azathioprine
- [ ] Ciamexone
- [ ] Cyclosporin
- [ ] Glucocorticoid therapy
- [ ] Intravenous immunoglobulin
- [ ] Methotrexate
- [ ] Mycophenolate
- [ ] Rituximab
- [ ] Selenium
- [ ] Somatostatin analogues
- [ ] Teprotumumab
- [ ] Tocilizumab
- [ ] Others (Please Specify) ______________________

When do you recommend glucocorticoid therapy for TED patients (tick as many as applicable)?*

- [ ] Early stages of TED
- [ ] Active phase of TED
- [ ] Moderate to Severe TED
- [ ] Rapidly progressive, including patients with significant motility deficits
- [ ] Compressive optic neuropathy
- [ ] Patients with progressive strabismus
- [ ] Recurrence of active TED
- [ ] Diplopia
- [ ] Enlargement of extraocular muscles
- [ ] Congestive symptom
- [ ] Corneal involvement
- [ ] Other (Please Specify) ______________________

What are your glucocorticoid therapy regimens?*

- [ ] IV methylprednisolone of 6 weekly doses of 500 mg then 6 weekly doses of 250 mg to a total dose of 4.5 g
- [ ] IV methylprednisolone 500 mg daily for 3 consecutive days per week for 2 weeks, followed by 250 mg daily for 3 consecutive days per week for another 2 weeks and by tapering oral prednisone
- [ ] IV methylprednisolone 500 -1000 mg alternate daily for 1 or 2 weeks, considering orbital decompression if present DON
- [ ] A high-dose oral prednisolone 100 mg daily reducing by 10mg per week
- [ ] Other (Please Specify) ______________________

What are the common improvement during follow-up (after 1-3 months period) after glucocorticoid therapy?*

- [ ] Spontaneous retrobulbar pain
- [ ] Gaze evoked orbital pain
- [ ] Eyelid erythema
- [ ] Conjunctival redness
- [ ] Eyelid swelling
- [ ] Inflammation of the caruncle or plica
- [ ] Increased of eye movements in any direction above 5 degreesIn
- [ ] Increased of visual acuity of more than 1 line on the Shelley chart
- [ ] Decreased of proptosis more than 2mm
- [ ] Chemosis
- [ ] Other (Please Specify) ______________________

What are the side effects during glucocorticoid therapy in courses to treat TED (tick as many as applicable)? *

- [ ] Weight gain
- [ ] Hypertension
- [ ] Depression
- [ ] Sleeplessness
- [ ] Hyperglycaemia/ Impaired fasting glucose (IFG)/ Impaired glucose tolerance (IGT)/ Diabetes mellitus (DM)
- [ ] Decreased BMD or osteoporosis
- [ ] Hyperlipidemia
- [ ] Impaired liver function
- [ ] Hypokalemia
- [ ] Hyperuricemia
- [ ] Other (Please Specify) ________________________________

What are the common improvements during follow-up (after 1-3 months period) after immunosuppressive agents(including cytostatic agents and mono-and polyclonal antibodies) therapy (tick as many as applicable)?*

- [ ] Spontaneous retrobulbar pain
- [ ] Gaze evoked orbital pain
- [ ] Eyelid erythema
- [ ] Conjunctival redness
- [ ] Eyelid swelling
- [ ] Inflammation of the caruncle or plica
- [ ] Decreased of eye movements in any direction above 5 degrees
- [ ] Decreased of visual acuity of more than 1 line on the Shelley chart
- [ ] Decreased of proptosis more than 2mm
- [ ] Chemosis
- [ ] Other (Please Specify) ________________________________

What are the side effects during immunosuppressive agents (including cytostatic agents and mono-and polyclonal antibodies) therapy in courses to treat TED? *

- [ ] Irreversible rise of the plasma creatinine
- [ ] More frequently hypertension
- [ ] Bone marrow suppression
- [ ] Nausea and vomiting
- [ ] Increased risk of infection
- [ ] Hyperglycemia in patients with diabetes
- [ ] Other (Please Specify) ____________________

Periorbital Injection

Do you treat TED with periorbital injection?*

- ○ Never
- ○ Sometimes
- ○ Always

What do you choose for periorbital injection of TED eyelid retraction (tick as many as applicable)?*

- [ ] Triamcinolone acetonide
- [ ] Dexamethasone
- [ ] Botulinum toxin
- [ ] Hyaluronic acid gel filler
- [ ] Other (Please Specify) ____________________

Please introduce the treatment strategy about injection of triamcinolone for the treatment of upper eyelid retraction associated with TED:*

Injection site:

- ○ Conjunctiva just superior to the upper tarsal edge
- ○ The upper eyelid was pulled upward , between the superior tarsal border and the superior fornix subconjunctival region of the eyelid between the conjunctiva and Müller’s muscle, along the upper border of the tarsus
- ○ Other (Please Specify) ____________________

*

Therapeutic doses?

- ◯ 10 mg
- ◯ 20 mg
- ◯ 30 mg
- ◯ 40 mg
- ◯ Other (Please Specify) ______________________

*

Time between each injection?

- ◯ 1 week
- ◯ 2 weeks
- ◯ 3 weeks
- ◯ 4 weeks
- ◯ Other (Please Specify) ______________________

*

Total number of injections?

- ◯ One
- ◯ Two
- ◯ Three
- ◯ Four
- ◯ More than four
- ◯ Other (Please Specify) ______________________

*

What are the side effects during triamcinolone acetone due injection therapy (tick as many as applicable)?

- ☐ Cataract
- ☐ Eyelid ecchymosis and/or subconjunctival hemorrhage
- ☐ Increased intraocular pressure
- ☐ Increase the psychological burden of patients
- ☐ Infection
- ☐ Menstrual changes
- ☐ Ptosis
- ☐ Raising blood pressure
- ☐ Raising blood sugar
- ☐ Swelling
- ☐ Vision loss
- ☐ Other (Please Specify) ________________

Please introduce the treatment strategy about intraorbital steroid injection for active TED*

Injection site：

- ○ Superior temporal quadrant
- ○ Inferior temporal quadrant
- ○ Superior nasal quadrant
- ○ Inferior nasal quadrant
- ○ Inferior quadrant
- ○ Other (Please Specify) ________________

*

Therapeutic doses?

- ○ 10 mg
- ○ 20 mg
- ○ 30 mg
- ○ 40 mg
- ○ Other (Please Specify) ________________

*

Time between each injection?

- ◯ 1 week
- ◯ 2 weeks
- ◯ 3 weeks
- ◯ 4 weeks
- ◯ Other (Please Specify) ____________________

*

Total number of injections?

- ◯ One
- ◯ Two
- ◯ Three
- ◯ Four
- ◯ More than four
- ◯ Other (Please Specify) ____________________

*

What are the side effects during periorbital corticosteroid injection therapy?

- ☐ Eyelid ecchymosis and/or subconjunctival hemorrhage was
- ☐ Increased intraocular pressure
- ☐ Cataract
- ☐ Vision loss
- ☐ Swelling
- ☐ Raising blood pressure
- ☐ Raising blood sugar
- ☐ Increase the psychological burden of patients
- ☐ Ptosis
- ☐ Infection
- ☐ Menstrual changes
- ☐ Other (Please Specify) ____________________

Please introduce the treatment strategy about Botoxs injection treatment for upper eyelid retraction associated with TED:*

Injection site：

- ◯ Transcutaneously
- ◯ Subconjunctival
- ◯ Other (Please Specify) ____________________

*

Therapeutic doses per point?

- ◯ 1 UI
- ◯ 1.5 UI
- ◯ 2 UI
- ◯ 3 UI
- ◯ 4 UI
- ◯ 5 UI
- ◯ Other (Please Specify) ____________________

*

How many points per injection?

- ◯ One
- ◯ Two
- ◯ Three
- ◯ Other (Please Specify) ____________________

*

Time interval between each injection?

- ◯ 1 month
- ◯ 2 months
- ◯ 3 months
- ◯ 4 months
- ◯ 5 months
- ◯ 6 months
- ◯ Other (Please Specify) ________________

*

What are the side effects of Botoxs injection treatment (tick as many as applicable)?

- ☐ Cataract
- ☐ Diplopia
- ☐ Lagophthalmos and ecchymosis at the injection site
- ☐ Increased intraocular pressure
- ☐ Increase the psychological burden of patients
- ☐ Infection
- ☐ Menstrual changes
- ☐ Ptosis
- ☐ Raising blood pressure
- ☐ Raising blood sugar
- ☐ Subconjunctival hemorrhage
- ☐ Other (Please Specify) ________________

Please introduce the treatment strategy about hyaluronic acid gel filler injection treatment for eyelid retraction associated with TED:*

Injection site:

- ◯ Upper eyelid
- ◯ Lower eyelid
- ◯ Transcutaneously
- ◯ Subconjunctival
- ◯ Other (Please Specify) ______________________

*

Therapeutic doses?

- ◯ 0.1 ml
- ◯ 0.2 ml
- ◯ 0.3 ml
- ◯ 0.4 ml
- ◯ 0.5 ml
- ◯ Other (Please Specify) ______________________

*

Time interval between each injection?

- ◯ 1 month
- ◯ 2 months
- ◯ 3 months
- ◯ 4 months
- ◯ 5 months
- ◯ 6 months
- ◯ Other (Please Specify) ______________________

*

What are the side effects of hyaluronic acid gel filler injection treatment?

- [ ] Erythema, edema, and ecchymosis at the injection site
- [ ] Contour irregularity and lumps, fluid build-up, and blue or dark color changes
- [ ] Cutaneous hypersensitivity reactions
- [ ] Branch retinal artery occlusion
- [ ] Eyelid ecchymosis and/or subconjunctival hemorrhage
- [ ] Vision loss
- [ ] Increased intraocular pressure
- [ ] Cataract
- [ ] Raising blood pressure
- [ ] Raising blood sugar
- [ ] Increase the psychological burden of patients
- [ ] Ptosis
- [ ] Infection
- [ ] Menstrual changes
- [ ] Other (Please Specify) ______________________

Radiotherapy

Do you treat TED with orbital radiotherapy (ORT)?*

- ○ Never
- ○ Sometimes
- ○ Always

When do you recommend orbital radiotherapy (ORT) for TED patients (tick as many as applicable)?*

- ☐ Early stages of TED
- ☐ Recurrence of TED
- ☐ Active phase of TED
- ☐ Inactive phase of TED
- ☐ Mild TED
- ☐ Moderate to Severe TED
- ☐ Rapidly progressive, including patients with significant motility deficits
- ☐ DON
- ☐ Corneal involvement
- ☐ Patients with progressive strabismus
- ☐ Congestive symptom
- ☐ Proptosis
- ☐ Eyelid retraction
- ☐ Lower eyelid entropion
- ☐ Diplopia
- ☐ Enlargement of extraocular muscles
- ☐ Cosmesis
- ☐ Poor therapeutic effect of steroid or immunosuppressant
- ☐ Other (Please Specify) ______________________

From your experience, which is the most common improvement during follow-up (after 1-3 months period) after orbital radiotherapy (ORT) (tick as many as applicable)?*

- ☐ Spontaneous retrobulbar pain
- ☐ Gaze evoked orbital pain
- ☐ Eyelid erythema
- ☐ Conjunctival redness
- ☐ Eyelid swelling
- ☐ Chemosis
- ☐ Inflammation of the caruncle or plica
- ☐ Increased of eye movements in any direction above 5 degrees
- ☐ Increased of visual acuity of more than 1 line on the Shelley chart
- ☐ Decreased of proptosis more than 2mm

Whether you use orbital radiotherapy in conjunction with corticosteroid therapy ?*

- ○ Yes
- ○ No

What are the complications of orbital radiotherapy (ORT)? *

- ☐ Cataract formation
- ☐ Radiation retinopathy
- ☐ Head and neck cancer caused by ORT
- ☐ None have been found so far
- ☐ Other (Please Specify) ____________________
