# Supplementary material for: Comparison Between Oculoplastic Surgeons and Two Large Language Models in the Nonsurgical Management of Thyroid Eye Disease: A Questionnaire‐Based Cross‐Sectional Study
Source: Scientifica (Cairo). 2026 Oct 14;2026:2900790. doi: 10.1155/sci5/2900790 (PMC13669656; doi:10.1155/sci5/2900790)
Supplement: Supplementary file 2 [file SCI5-2026-2900790-s001.docx]

Supplementary Table 1. Characteristics of participating physicians.

| Physicians | Practice in Oculoplastics | Managed TED in total (n) | Country of Practice |
| --- | --- | --- | --- |
| Human1 | <10 years | 100~500 | Indonesia |
| Human2 | 10~20 years | 100~500 | Indonesia |
| Human3 | 10~20 years | 10~100 | Indonesia |
| Human4 | 10~20 years | 100~500 | China’s mainland |
| Human5 | 20~30 years | 10~100 | Philippines |
| Human6 | <10 years | 500~1000 | South Korea |
| Human7 | >30 years | 500~1000 | India |
| Human8 | 20~30 years | >1000 | Japan |
| Human9 | 10~20 years | >1000 | China’s mainland |
| Human10 | 10~20 years | 100~500 | Vietnam |
| Human11 | <10 years | <10 | Philippines |
| Human12 | 10~20 years | 10~100 | Myanmar |
| Human13 | 20~30 years | 100~500 | South Korea |
| Human14 | 20~30 years | >1000 | Philippines |
| Human15 | 10~20 years | >1000 | South Korea |
| Human16 | 20~30 years | >1000 | China’s mainland |
| Human17 | 10~20 years | >1000 | China’s mainland |

*Values for mean Jaccard and Cosine represent the average similarity between each physician’s responses and the outputs generated by the respective LLMs.
“Practice in Oculoplastics” indicates the total years of one’s practice in oculoplastic subspecialty. “Managed TED (n)” denotes the approximate number of TED cases one has managed in career.

Supplementary Figure 1. Overall consistency between physicians and LLMs.
